# Explainable Machine Learning Reveals Pattern-Specific Drivers of Depression in Middle-Aged and Older Adults with Multimorbidity: A Nationwide Cross-Sectional Study from CHARLS

**DOI:** 10.3390/healthcare14152341

**Published:** 2026-08-01

**Authors:** Yongze Zhao, Munhin Choi, Nansi Li, Qingyu Qiao, Junming Chen, Zhaokai Huang, Ying Bian

**Affiliations:** 1 State Key Laboratory of Mechanism and Quality of Chinese Medicine, University of Macau, N22 Building, Avenida da Universidade, Taipa, Macau SAR 999078, China; 2 Institute of Chinese Medical Sciences, University of Macau, N22 Building, Avenida da Universidade, Taipa, Macau SAR 999078, China; 3 Department of Public Health and Medicinal Administration, Faculty of Health Sciences, University of Macau, E12 Building, Avenida da Universidade, Taipa, Macau SAR 999078, China; 4 Faculty of Health and Wellness, City University of Macau, Avenida Padre Tomás Pereira, Taipa, Macau SAR 999078, China; 5 Department of Language Science and Technology, Faculty of Humanities, The Hong Kong Polytechnic University, Kowloon, Hong Kong SAR 999077, China; 6 Peking University First Hospital, No. 8 XiShiku Street, Xicheng District, Beijing 100034, China

**Keywords:** depressive symptoms, multimorbidity patterns, machine learning, Shapley Additive exPlanations (SHAP)

## Abstract

**Background/Objectives:** Depression is a leading cause of disability among middle-aged and older adults, with its burden markedly amplified by multimorbidity. In rapidly aging China, distinct multimorbidity phenotypes interact with depressive symptoms through complex, non-linear pathways that traditional models struggle to capture. Interpretable machine learning offers high predictive accuracy alongside clinically transparent explanations, yet few studies have applied this approach to large national Chinese cohorts while stratifying by empirically derived multimorbidity patterns. The aim of this study was to identify the pattern-specific determinants of depression among middle-aged and older adults with multimorbidity using explainable machine learning techniques based on data from the China Health and Retirement Longitudinal Study (CHARLS). **Methods:** In this cross-sectional analysis of the China Health and Retirement Longitudinal Study (CHARLS) 2020 data, 14,981 participants were divided into a multimorbidity group (≥2 of 12 physician-diagnosed chronic conditions; n = 8265) and a matched control group (0 or 1 condition; n = 6716). Depressive symptoms were defined as a CES-D-10 score ≥ 10 (binary outcome). Forty-four candidate predictors were screened via LASSO regression, the Boruta algorithm, and univariate logistic regression. Fifteen supervised machine learning models were trained separately for each group using SMOTE for class imbalance, nested 5-fold cross-validation for hyperparameter tuning, and an independent 20% test set for external validation. Model performance was ranked by the composite AB Score (equal weighting of AUROC and Brier score). The champion models were interpreted globally via SHAP summary plots, directional waterfall diagrams, and novel impact-intensity network diagrams. The same pipeline was repeated across four literature-derived multimorbidity patterns within the multimorbidity group. **Results:** Depressive-symptom prevalence was 42.96% (3551/8265) in the multimorbidity group versus 27.01% (1814/6716) in the control group. The Gaussian process classifier achieved the highest AB Score (0.7782) in the multimorbidity group (external AUROC of 0.7545, Brier score of 0.1981); the generalized additive model was optimal in the control group (AB Score 0.7856, external AUROC 0.7387, Brier score 0.1676). Across both groups, the top contributors by mean absolute SHAP were IADL disability, abnormal sleep duration, ADL disability, rural residence, and lower education level. Pattern-specific SHAP networks revealed clinically meaningful heterogeneity: cardio-metabolic clusters were driven by marital status and headache; digestive–joint clusters by IADL disability and shoulder–knee pain synergy; respiratory clusters by gender and multisite pain; and cardiovascular–digestive clusters by sleep duration and vigorous activity. **Conclusions:** Interpretable machine learning models demonstrated clinically useful discrimination and excellent calibration while revealing universal drivers (functional disability and sleep disturbance) and cluster-specific synergistic interactions underlying depressive symptoms in multimorbid Chinese adults. These phenotype-tailored insights provide a practical roadmap for precision screening and targeted interventions in aging populations.

## 1. Introduction

Depression ranks among the leading causes of disability-adjusted life years (DALYs) worldwide, with particularly profound effects on middle-aged and older adults [1]. In China, the world’s most rapidly aging country, recent national surveys estimate the prevalence of depressive symptoms among adults aged 45 and older to be between 25% and 40%, imposing substantial personal, familial, and societal burdens [2]. Concurrently, multimorbidity—the coexistence of two or more chronic conditions—has become a defining characteristic of aging populations. National data indicate that over half of Chinese adults aged 60 and older live with multimorbidity [3], a condition associated not only with accelerated functional decline, higher healthcare expenditures, and increased mortality but also with a markedly elevated risk of depressive symptoms. The synergistic interaction between physical chronic diseases and mental health arises through shared pathways, including chronic inflammation, pain, social isolation, reduced physical activity, and disrupted sleep; however, the precise mechanisms remain incompletely understood [4,5]. When patients grapple with multiple chronic conditions concurrently, their psychological burden extends far beyond physical limitations. The relentless strain of symptoms erodes one’s sense of personal agency and self-efficacy, fostering maladaptive cognitive patterns such as catastrophizing, rumination, and learned helplessness. These distortions heighten systemic emotional vulnerability, independently lowering the threshold for severe depressive episodes and creating self-reinforcing cycles. This underscores the necessity of integrated biopsychosocial interventions targeting both somatic management and cognitive–emotional resilience.

Emerging evidence suggests that distinct multimorbidity phenotypes—such as cardio-metabolic, digestive–joint, respiratory, and cardiovascular–digestive clusters—exhibit unique associations with depressive symptoms [6]. However, most prior research has relied on traditional logistic regression or structural equation modeling, which struggle to capture high-dimensional interactions, non-linear effects, and individual-level heterogeneity. Interpretable machine learning offers distinct advantages in this context: ensemble- and kernel-based algorithms can model complex, non-linear relationships and high-order interactions among dozens of predictors without strong parametric assumptions, and stratification by data-driven multimorbidity patterns allows detection of heterogeneous mechanisms that would be averaged out in conventional regression. These properties make interpretable ML particularly suitable for generating actionable, phenotype-specific insights from large-scale observational data such as CHARLS, while maintaining transparency required for clinical and policy translation.

Interpretable machine learning approaches address these limitations by providing both high predictive accuracy and clinically transparent explanations [7]. Techniques such as SHapley Additive exPlanations (SHAP) enable quantification of each predictor’s contribution and synergistic interactions, thereby bridging the gap between statistical prediction and actionable clinical insights [8]. To date, however, few studies have applied systematic, interpretable machine learning frameworks to large national survey samples in China, and none have stratified analyses by empirically derived multimorbidity patterns while simultaneously comparing multimorbid and non-multimorbid populations.

Compared with recent SHAP-based explainable machine learning studies on depression and multimorbidity or chronic diseases [9,10], the present work offers several distinct advances. We performed explicit stratification by four empirically derived multimorbidity phenotypes with dedicated modeling in each subgroup, included a matched non-multimorbid control group for direct contrast, and introduced impact-intensity network diagrams to visualize synergistic interactions. In addition, we employed a composite AB Score balancing discrimination and calibration for model selection, identified non-parametric models (GPC and GAM) as top performers, and translated findings into China-specific equity-focused policy recommendations addressing rural structural disparities. These elements collectively advance interpretable machine learning toward precision public mental health in aging populations.

The present study addresses these critical gaps by leveraging the 2020 wave of the China Health and Retirement Longitudinal Study (CHARLS), a large, national cohort of middle-aged and older Chinese adults. We developed and rigorously validated 15 supervised machine learning models separately for participants with multimorbidity (≥2 of 12 physician-diagnosed chronic conditions) and a matched control group (0 or 1 condition). Feature selection combined LASSO regression, the Boruta algorithm, and univariate logistic screening with variance inflation factor checks to ensure parsimony and the absence of multicollinearity. Models were evaluated using a composite AB Score (equal weighting of AUROC and 1–Brier score), nested cross-validation, and decision-curve analysis for clinical utility. The champion models were interpreted via global SHAP summary plots, directional waterfall diagrams, and novel impact-intensity network diagrams that visualize synergistic interactions. To further elucidate heterogeneity, we repeated the pipeline across four literature-derived multimorbidity patterns. By integrating class balancing (SMOTE), standardized preprocessing, and transparent interpretability, this work provides robust, phenotype-specific evidence to guide precision public mental health strategies in aging China and comparable rapidly aging populations worldwide. The aim of this study was to identify the pattern-specific determinants of depression among middle-aged and older adults with multimorbidity using explainable machine learning techniques based on data from the China Health and Retirement Longitudinal Study (CHARLS).

## 2. Materials and Methods

### 2.1. Data Source and Study Population

This cross-sectional study utilized data from the China Health and Retirement Longitudinal Study (CHARLS). The detailed study design and sampling methods have been reported previously [11,12]. In brief, CHARLS is a nationally representative survey among middle-aged and older individuals from 150 counties/districts and 450 villages/urban communities in 28 provinces. The study sample comprised households employing a multi-stage stratified probability-proportional-to-size (PPS) sampling technique with face-to-face interviews and physical measurements. For each participant, study variables were repeatedly measured at every available time point from 1 January 2011, to 31 December 2020. The present analysis focused exclusively on the 2020 cross-sectional wave (wave 5) to examine associations between multimorbidity patterns and depressive symptoms. The CHARLS targets community-dwelling middle-aged and older adults aged 45 years and older (and their spouses). All methods concerning human participants in our study were conducted following the ethical standards laid out in the 1964 Declaration of Helsinki and its subsequent amendments. Ethical approval for all waves of the CHARLS was obtained from the Institutional Review Board (IRB) at Peking University [11]. The IRB approval number for the main household survey, including anthropometrics, is IRB00001052-11015. Written signed informed consent was obtained at recruitment from all participants, including legal representatives for illiterate participants. All data in this study were aggregated at the regional level and did not contain any identifiable information at the individual or household levels. Therefore, no specific ethical issues were raised. The study followed the Strengthening the Reporting of Observational Studies in Epidemiology (STROBE) guidelines for cross-sectional studies (see Supplementary Table S1).

Participants were divided into two mutually exclusive groups based on the presence of 12 self-reported physician-diagnosed chronic conditions: hypertension, diabetes, dyslipidaemia, arthritis, lung diseases, kidney diseases, liver diseases, cancer, heart disease, stroke, stomach diseases, and asthma.

Participants were selected from the CHARLS 2020 wave according to the following criteria. Inclusion criteria were: (1) community-dwelling middle-aged and older adults aged 45 years and older (and their spouses); (2) participation in the CHARLS 2020 (wave 5) cross-sectional survey; (3) completion of the CES-D-10 depression scale; and (4) provision of complete data on the 12 chronic conditions and key covariates. Exclusion criteria were: (1) age less than 45 years; (2) presence of other mental disorders; (3) presence of memory-related diseases; and (4) missing data on depressive symptoms (CES-D-10) or key covariates.

**Figure 1:**
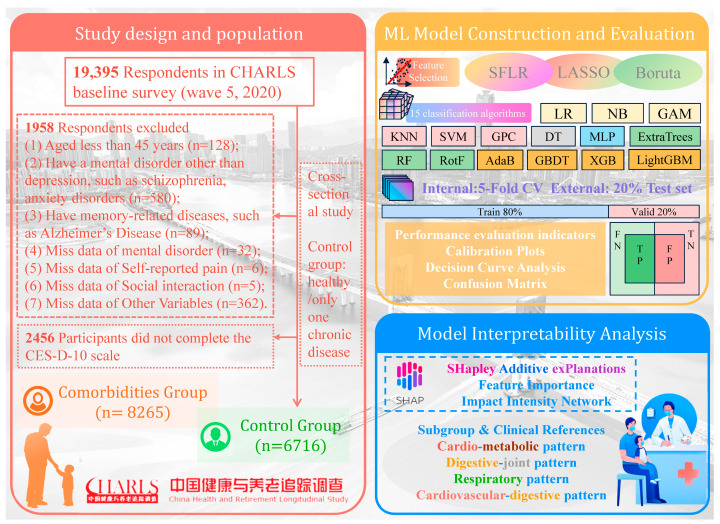
Study flow diagram. Flowchart illustrating participant selection from the China Health and Retirement Longitudinal Study (CHARLS) 2020 wave, group division, and machine learning modeling pipeline. A total of 14,981 middle-aged and older adults (aged ≥45 years) were included after excluding individuals with missing data on depressive symptoms or key covariates. Participants were stratified into the multimorbidity group (≥2 of 12 chronic conditions; n = 8265) and the control group (0 or 1 condition; n = 6716). Each group was randomly split into a training set (80%) for nested 5-fold stratified cross-validation and hyperparameter tuning, and an independent test set (20%) for external validation. Fifteen supervised machine learning models were trained separately in each group.

After applying these criteria, 14,981 participants remained in the final analytic sample and were divided into two groups: the multimorbidity (comorbidities) group included individuals with two or more of these conditions (n = 8265); and the control group comprised participants with zero or one condition (n = 6716). To improve comparability between groups, we performed post hoc balancing on age and gender (as shown in Table 1); formal 1:1 propensity score matching was not applied because the primary scientific interest was in the overall multimorbid population rather than a narrowly matched subset, and the large sample size allowed sufficient power for stratified analyses. Individuals with missing data on depressive symptoms or key covariates were excluded. There were no missing values in either the control group or the multimorbidity group; therefore, no additional handling was required. The final analytic sample consisted of 14,981 participants. Figure 1 is the flow chart showing how we constructed the study design.

### 2.2. Outcome Definition

Depressive symptoms were assessed using the Center for Epidemiologic Studies Depression Scale (CES-D-10), which was highly validated for use in general populations and indicated adequate reliability and validity for the community-dwelling older population in China [13,14]. The CES-D-10 includes 10 questions with 4 response options: (1) None or almost none (less than 1 day); (2) Rare (1–2 days); (3) Frequent (3–4 days); and (4) Almost always (5–7 days), reflecting the participant’s experiences over the past week, such as feeling bothered, having trouble concentrating, feeling depressed, and feeling hopeful about the future. The total score ranges from 0 to 30. A cutoff score  ≥  10 was used to identify the respondents who had depressive symptoms, following the routine practice in the existing literature [15,16]. The outcome was treated as a binary variable (1 = depressive symptoms, 0 = no depressive symptoms) for all machine learning analyses.

### 2.3. Research Covariates

Based on previous studies [17,18] and expert opinions (two independent geriatricians from Peking University Affiliated Hospitals), a total of 44 potential predictor variables were initially selected from the CHARLS questionnaire and harmonized for analysis. Candidate covariates included: (1) Demographic characteristics: age, gender, education level, marital status, rural/urban residence, and retirement status; (2) socioeconomic factors: medical insurance, pension insurance, family size, and number of living children; (3) functional status: Activities of Daily Living (ADL, 6 items), Instrumental Activities of Daily Living (IADL, 5 items); (4) health behaviors: sleep duration, physical exercise (vigorous/moderate/mild), alcohol consumption, and smoking (ever smoked/current smoking); (5) chronic disease history: The 12 chronic diseases listed in Section 2.1; (6) pain sites: 15 locations (headache, shoulder pain, arm pain, wrist pain, finger pain, chest pain, stomach pain, back pain, lower back pain, hip pain, leg pain, knee pain, ankle pain, toe pain, and neck pain); and (7) social activities: 8 types (visiting friends, playing cards, volunteering, dancing/exercising, club activities, volunteer/charity work, training courses, and other social activities). The final set of predictors entering the models was determined through a variable selection process. All 12 chronic conditions were ascertained through self-reported physician diagnosis using the standard CHARLS questionnaire items (e.g., “Have you been diagnosed by a doctor with [condition]?”). The exact wording of each question is provided in Supplementary Table S2.

### 2.4. Statistical Analysis

Descriptive statistics are presented first to provide an overview of the baseline characteristics of the study participants. Continuous variables are expressed as mean ± standard deviation (SD), while categorical variables are presented as frequencies and percentages. Analyses were conducted separately for the multimorbidity and control groups, with each group further subdivided into depression-negative and depression-symptom subgroups. Differences were analyzed using t-tests and Pearson’s chi-square tests.

Stata software (version 17.0, StataCorp LLC, College Station, TX, USA) and R (version 4.5.2) were used for descriptive statistics and feature screening. All data preprocessing, model training, evaluation, and visualization were performed in Python 3.14 using pandas, NumPy, scikit-learn, XGBoost, LightGBM, PyGAM, and SHAP libraries. Figures were generated with Matplotlib (version 3.11.1) and Seaborn (version 0.13.2). A significance level of α = 0.05 (two-sided) was used for baseline comparisons.

### 2.5. Feature Screening

To identify the most relevant predictors and reduce dimensionality while avoiding multicollinearity, three complementary methods were applied independently to each group.

(1) Univariate logistic regression: Each variable was tested individually for association with depressive symptoms, retaining those with *p* < 0.05. (2) Least Absolute Shrinkage and Selection Operator (LASSO) regression: LASSO regression improves model refinement by applying a penalty function that shrinks specific regression coefficients, thereby imposing a constraint on the sum of their absolute values to remain below a predetermined threshold [19]. We utilize the glmnet (4.1-10) in R for LASSO regression. Ten-fold cross-validation was used to select the optimal penalty parameter λ, with the lambda.1se criterion (the most parsimonious model within one standard error of the minimum), and (3) the Boruta algorithm: A random forest-based all-relevant feature selection method that identifies variables truly associated with the outcome. It assesses the importance of each feature by creating to the original variables in the dataset [20]. Boruta (Version 9.0.0) in R was utilized to conduct feature selection. This algorithm iteratively compares the importance of each original variable with that of its corresponding shadow variable, assessing variable importance over 200 iterations or until all variables reach stability. Variables selected by all three methods were retained via Venn diagram intersection. This stringent intersection approach was adopted to enhance robustness by requiring consensus across parametric (LASSO), tree-based all-relevant (Boruta), and classical statistical methods, thereby promoting parsimony and reproducibility. To prevent data leakage, the independent 20% external test set was isolated prior to any feature selection or modeling; all screening steps were performed exclusively on the remaining 80% training data used for nested cross-validation. This stringent intersection strategy ensures reproducibility and guards against method-specific artifacts, at the acceptable cost of potentially conservative feature sets. Before selection, all continuous variables were standardized, and variance inflation factor (VIF) analysis confirmed no multicollinearity (all VIF < 5).

### 2.6. Machine Learning Models Construction and Validation

For scale-sensitive models (LR, KNN, SVM, MLP, and GPC), all continuous features were standardized using StandardScaler (zero mean, unit variance), while tree-based models and GAM used raw features. Categorical variables were encoded using one-hot encoding. The prevalence of depression in both the multimorbidity group and the control group among the participants in this study exhibits class imbalance. This imbalance tends to bias the accuracy of machine learning models used to predict the probability of depressive symptoms toward the more prevalent class. To mitigate this issue, we employed the Synthetic Minority Over-sampling Technique (SMOTE) to balance the classes in the dataset [21]. A consistent preprocessing pipeline was implemented across all models to guarantee equitable comparability. Although class imbalance was moderate (depression prevalence approximately 43% in the multimorbidity group and 27% in the control group), SMOTE was applied consistently across all 15 models to ensure robust probability calibration and to prevent bias toward the majority class, particularly in tree-based, kernel, and neural network algorithms.

SMOTE was selected after preliminary comparisons as it offered the optimal balance between minority-class sensitivity and calibration preservation. Sensitivity analyses without oversampling (Supplementary Tables S7 and S8) yielded consistent champion models and feature rankings, confirming robustness; alternative approaches (e.g., class weighting, Borderline-SMOTE) produced comparable or marginally inferior AB Scores.

To comprehensively evaluate both linear and non-linear relationships while balancing predictive performance and interpretability, we developed fifteen supervised machine learning models separately for each group (multimorbidity and control): logistic regression (LR), k-nearest neighbors (KNN), naïve Bayes (NB), decision tree (DT), support vector machine (SVM), multilayer perceptron (MLP), Gaussian process classifier (GPC), random forest (RF), extra trees, AdaBoost, gradient boosting decision tree (GBDT), XGBoost, LightGBM, rotation forest (RotF), and generalized additive model (GAM). These models were deliberately chosen to span diverse algorithmic families: traditional statistical (LR as explicit benchmark), instance-based (KNN), probabilistic (NB), tree-based ensembles (RF, Extra Trees, AdaBoost, GBDT, XGBoost, LightGBM, RotF), kernel methods (SVM, GPC), neural networks (MLP), and flexible non-parametric (GAM). This diversity allows rigorous head-to-head comparison of discrimination, calibration, and clinical utility.

The dataset was first randomly split into a training set (80%) and an independent test set (20%) using stratified sampling to preserve class balance (the prevalence of depression is the same in both the training set and the test set). All feature selection steps (LASSO, Boruta, and univariate logistic regression with VIF check) were performed exclusively on the training data to prevent information leakage from the test set. Nested stratified cross-validation was then applied exclusively within the training set for hyperparameter optimization and internal validation, while the held-out test set provided unbiased external validation. Specifically, an internal 5-fold stratified cross-validation integrated grid search and random search methods to optimize hyperparameters according to multiple evaluation metrics (AUROC, Brier score, etc.), whereas the independent test set evaluated model consistency and mitigated overfitting. This workflow ensures that feature selection and model tuning do not leak information from the test set.

Model discrimination was quantified by the area under the receiver operating characteristic curve (AUROC). Calibration was assessed using the Brier score and calibration plots. Traditionally, the performance of machine learning models is assessed using the AUC-ROC or calibrated AUC. Building on existing methodological advancements [22], we used the composite AB Score, calculated as follows:

$$AB\;Score=\;\alpha \times \bar{AUROC}+\left(1-\alpha \right)\times \left(1-\bar{Brier}\right)$$

This metric considers both discriminatory power and calibration. In this study, we set α = 0.5, thereby assigning equal weight to both aspects. The AB Score serves as the primary ranking metric for selecting the optimal model. Additional metrics included sensitivity, specificity, precision, F1-score, accuracy, and Youden’s index at the optimal threshold (maximizing Youden’s J). The model with the highest average AB Score (internal + external) was selected as the champion for each group. All metrics were computed separately for internal validation (5-fold cross-validation) and external validation (test set).

### 2.7. Model Interpretation and Clinical Utility

To ensure clinical interpretability, confusion matrices and calibration plots evaluated model reliability, while decision curve analysis (DCA) evaluated clinical net benefit across threshold probabilities. SHAP (SHapley Additive exPlanations) values further elucidated feature contributions and were computed for the best-performing model in each group. For tree-based models, TreeExplainer was used; for GPC and GAM, KernelExplainer with a logit link was applied. Global importance was visualized via summary dot plots and mean absolute SHAP bar plots.

Directional effects were illustrated with a custom vertical waterfall plot of average SHAP contributions. Feature interactions were depicted using custom circular impact-intensity network diagrams. In these visualizations, node size and color intensity are proportional to the mean absolute SHAP value, representing each feature’s global contribution to the model’s predictions. Edge thickness and color intensity represent the strength of pairwise synergistic interactions, derived from SHAP interaction values (directly available for tree-based models via TreeExplainer) or approximated through pairwise SHAP dependence plots or Spearman correlation of per-instance SHAP values when the explainer did not provide an explicit interaction matrix (as for GPC and GAM). This approach allows intuitive visualization of how features jointly influence depressive symptom risk beyond their main effects. A detailed description and rationale for this visualization method have been added to enhance transparency and reproducibility.

For the champion non-parametric models (Gaussian process classifier and generalized additive model), where native tree-based interaction matrices are unavailable, pairwise feature interactions were approximated using two complementary approaches. First, SHAP dependence plots (implemented via the shap Python library) were generated to visualize non-additive effects and potential interactions. Second, and more directly for the impact-intensity network diagrams, edge thickness was scaled proportionally to the absolute Spearman rank correlation coefficient (ρ) between the per-sample SHAP value vectors of each feature pair. This Spearman-based proxy captures the degree to which one feature’s marginal contribution covaries with another, serving as a computationally feasible surrogate for interaction strength when exact SHAP interaction values cannot be obtained. Edges were displayed only when |ρ| > 0.1 to reduce visual clutter while retaining meaningful synergies. All computations were performed in Python 3.13.9 using the following libraries: shap (for SHAP and dependence plots), scipy.stats (spearmanr function), numpy and pandas (for data handling), and networkx (for graph construction and circular layout). This transparent, reproducible workflow ensures that synergistic effects beyond main effects can be quantified and visualized even for non-tree models.

Within the multimorbidity group, four literature-derived multimorbidity patterns were examined [6]: (A) cardio-metabolic, (B) digestive–joint, (C) respiratory, and (D) cardiovascular–digestive. For each pattern, the same pipeline (model training, model evaluation, and SHAP analysis) was repeated, focusing on the impact-intensity network to reveal pattern-specific drivers of depressive symptoms.

## 3. Results

### 3.1. Participant Characteristics

A total of 14,981 middle-aged and older Chinese adults were included in the final analysis, comprising 8265 participants in the multimorbidity (comorbidities) group and 6716 in the control group (single disease or healthy). The prevalence of depressive symptoms (CES-D-10 positive) was 42.96% (3551/8265) in the multimorbidity group, significantly higher than the 27.01% (1814/6716) in the control group.

Participant characteristics in the 2020 wave are shown in Table 1. In both groups, participants with depressive symptoms were significantly older, more likely to be female, rural residents, less educated, unmarried or non-cohabitating, not yet retired, and had higher rates of ADL and IADL disabilities, abnormal sleep duration, current smoking, drinking, hip fracture, and most individual chronic conditions (hypertension, dyslipidaemia, arthritis, etc.; all *p* < 0.05). These differences were more pronounced in the multimorbidity group.

### 3.2. Feature Selection

**Figure 2:**
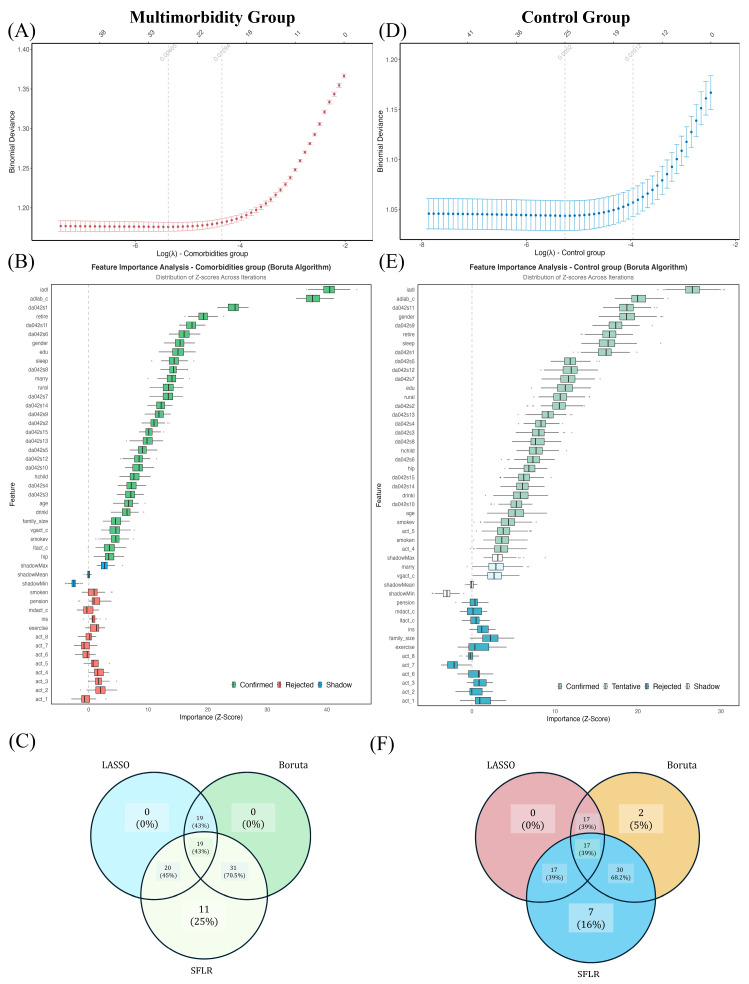
Feature selection results. (**A**,**D**) Factor screening based on the LASSO regression model, with the left dashed line indicating the best lambda value for the evaluation metrics (lambda. min) and the right dashed line indicating the lambda value for the model where the evaluation metrics are in the range of the best value by one standard error (lambda.1se); (**B**,**E**) box-and-whisker plot of variable selection results based on the Boruta algorithm; (**C**,**F**) venn diagrams showing variable selection based on Boruta, LASSO and univariate logistic regression. A total of 19 variables were selected for the multimorbidity group, and 17 variables for the control group.

The results of variable selection using LASSO regression (λ.1se), the Boruta algorithm, and univariate logistic regression are shown in Figure 2. In the multimorbidity group, LASSO selected 20 variables, Boruta selected 31, and univariate logistic regression selected 42, with a final set of 19 variables identified by Venn diagram intersection. In the control group, LASSO, Boruta, and univariate logistic regression selected 17, 32, and 37 variables, respectively, resulting in 17 final variables. All selected variables passed variance inflation factor (VIF) testing, indicating no significant multicollinearity.

The selected features common to both the multimorbidity group and control group included sociodemographic factors (education, gender, marital status, rural residence, and retirement), functional status (IADL and ADL disability), lifestyle (vigorous physical activity, alcohol consumption, and sleep duration), and pain-related variables (headache, shoulder pain, stomach pain, lower back pain, leg pain, and knee pain. The multimorbidity group additionally included hip fractures, chest pain, and toe pain. The control group additionally included finger pain. Complete lists are provided in Supplementary Tables S3 and S4.

### 3.3. Machine Learning Model Performance

**Figure 3:**
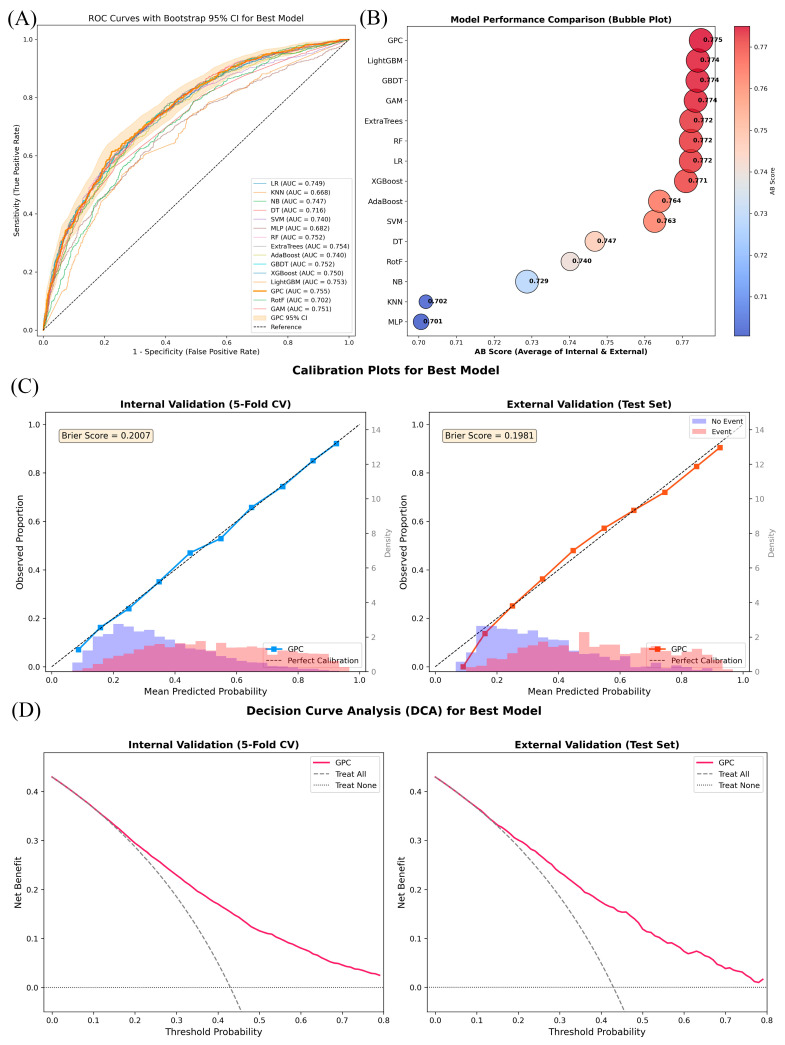
Performance of 15 machine learning models in the multimorbidity group. (**A**) Receiver operating characteristic (ROC) curves with bootstrap 95% confidence intervals for the 15 models on the independent test set. (**B**) Bubble plot ranking all models by AB Score (primary ranking metric); bubble size reflects external validation AUROC. (**C**) Calibration curves with histograms showing predicted probability distributions for negative (blue) and positive (red) classes. (**D**) Decision curve analysis (DCA) demonstrating net clinical benefit across threshold probabilities.

Fifteen interpretable machine learning models were trained and evaluated using 5-fold stratified cross-validation (internal) and an independent 20% test set (external). Model performance was assessed using multiple metrics, including AB Score (a weighted average of AUC and Brier score), AUROC, Brier score, F1 score, sensitivity, and specificity. As shown in Figure 3A–D, the Gaussian process classifier (GPC) achieved the best overall performance (AB Score = 0.7782) in the multimorbidity group. External validation AUROC was 0.7545 (95% CI via bootstrap shown in the ROC curve), with excellent calibration (Brier score = 0.1981) and net clinical benefit across threshold probabilities in decision curve analysis. At the optimal Youden threshold (0.4622), sensitivity was 0.6141, specificity 0.7762, precision 0.6739, and F1-score 0.6426. The bubble plot (Figure 3B) ranked all models by AB Score, confirming GPC superiority. The custom confusion matrix and probability distribution histogram further demonstrated clear separation between depressed and non-depressed participants.

**Figure 4:**
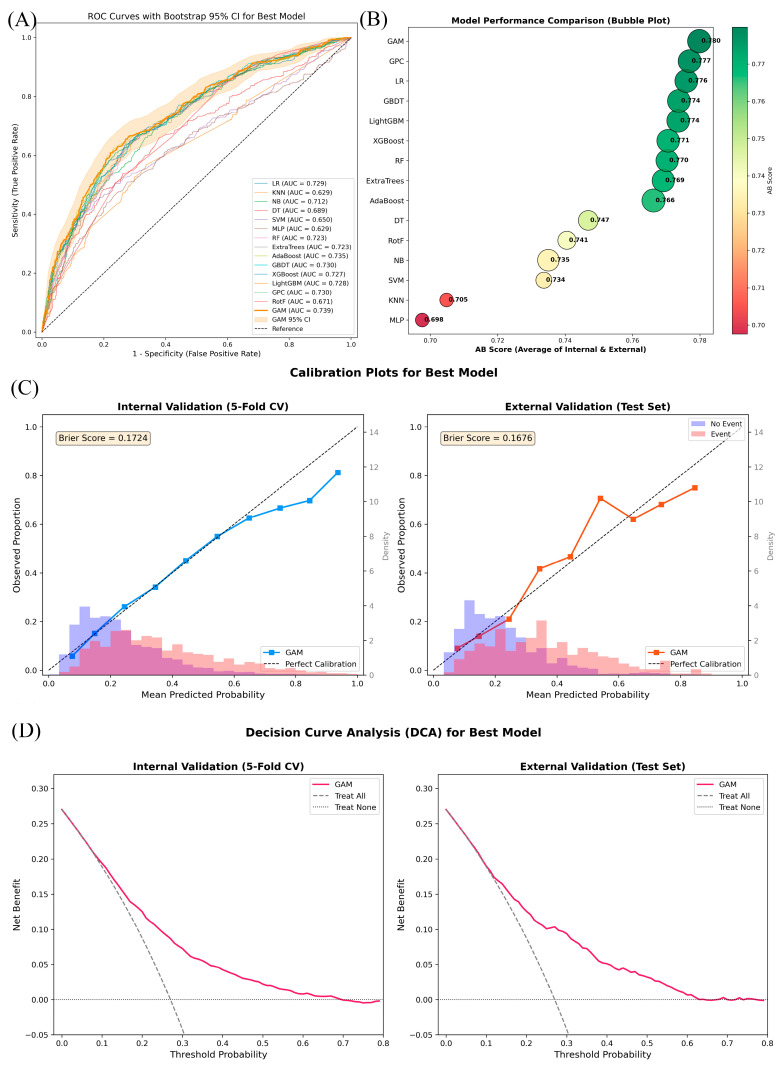
Performance of 15 machine learning models in the control group. (**A**) Receiver operating characteristic (ROC) curves with bootstrap 95% confidence intervals for the 15 models on the independent test set. (**B**) Bubble plot ranking all models by AB Score (primary ranking metric); bubble size reflects external validation AUROC. (**C**) Calibration curves with histograms showing predicted probability distributions for negative (blue) and positive (red) classes. (**D**) Decision curve analysis (DCA) demonstrating net clinical benefit across threshold probabilities.

In the control group, the generalized additive model (GAM) was the champion model (AB Score = 0.7856; Figure 4A–D). External AUROC reached 0.7387, Brier score was 0.1676, and the model provided robust net benefit in DCA. At the optimal threshold (0.2990), sensitivity was 0.6088, specificity 0.7788, precision 0.5046, and F1-score 0.5518. All 15 models showed acceptable discrimination (most AUROCs > 0.70), with close alignment between internal and external metrics indicating minimal overfitting. Supplementary Figure S1 shows the confusion matrix for the comorbidity group and control group. Complete performance metrics for all 15 models (AUROC, Brier score, sensitivity, specificity, precision, F1-score, accuracy, and Youden’s index) under both internal cross-validation and external validation are provided in Supplementary Tables S5 and S6.

To address potential concerns regarding the use of SMOTE, we conducted a sensitivity analysis by re-training all 15 models on the original unbalanced data. As shown in Supplementary Tables S7 and S8, model performance (AUROC, Brier score, sensitivity, specificity, and F1-score) remained largely consistent between the SMOTE and non-SMOTE approaches in both groups. The champion models (GPC in the multimorbidity group and GAM in the control group) retained their top ranking, and the overall conclusions regarding feature importance were unchanged. These results support the robustness of our primary findings.

Figure 3 and Figure 4 model abbreviations: LR = Logistic regression; KNN = K-Nearest neighbors; NB = Naïve Bayes; DT = Decision tree; SVM = Support vector machine; MLP = Multilayer perceptron; GPC = Gaussian process classifier; RF = Random forest; ExtraTrees = Extra trees; AdaBoost = Adaptive boosting; GBDT = Gradient boosting decision tree; XGBoost = Extreme gradient boosting; LightGBM = Light gradient boosting machine; RotF = Rotation forest; GAM = Generalized additive model. The champion model was GPC in the multimorbidity group and GAM in the control group.

### 3.4. Model Interpretability

**Figure 5:**
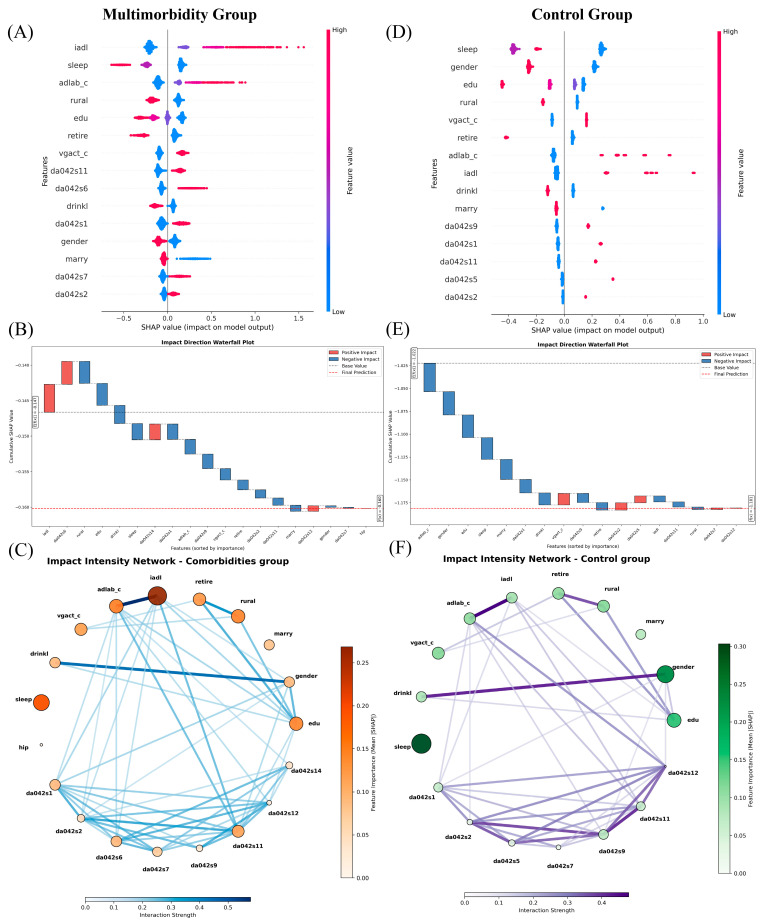
SHAP-based interpretability of optimal models. Global feature importance and interactions for the best-performing models (Gaussian process classifier in the multimorbidity group; generalized additive model in the control group). (**A**,**D**) SHAP summary dot plots showing the distribution and direction of feature contributions. Red dots indicate higher feature values; blue dots indicate lower values. (**B**,**E**) Directional waterfall plots illustrating the cumulative impact of all features on the model output relative to the baseline expectation. (**C**,**F**) Impact-intensity network diagrams visualizing synergistic interactions. Node size and color intensity represent mean |SHAP| (global importance). Edge thickness and color represent interaction strength (derived from SHAP interaction values for tree-based models or approximated via pairwise SHAP dependence/Spearman correlation for GPC and GAM; see Methods 2.7 for details). Strongest interactions are highlighted (e.g., IADL–ADL disability synergy).

SHAP analysis (Figure 5A–C) revealed the global feature importance and directional effects in the multimorbidity group (best model: GPC). The top five contributors by mean absolute SHAP value were: IADL disability (0.2687), sleep duration (0.2027), ADL disability (0.1507), rural residence (0.1454), and education level (0.1438). The directional waterfall plot showed that greater IADL/ADL disability, shorter/longer sleep duration, rural residence, and lower education consistently increased predicted depression risk (positive SHAP contributions), while vigorous activity and certain drinking patterns were protective. The impact intensity network diagram highlighted the strongest synergistic interactions between IADL and ADL disability (interaction strength of 0.5788), followed by gender–drinking (0.4751) and rural–retirement (0.4065).

In the control group (best model: GAM, Figure 5D–F), the leading features were sleep duration (0.3035), gender (0.2368), education (0.1601), rural residence (0.1168), and vigorous activity (0.1168). Negative SHAP values for higher education, male gender, and vigorous activity indicated protective effects, whereas ADL disability, rural residence, and pain symptoms (especially lower back and leg pain) increased risk. The network diagram underscored strong interactions between IADL–ADL disability (interaction strength 0.4786) and gender–drinking (0.4352).

### 3.5. Subgroup Analysis of Multimorbidity Patterns

**Figure 6:**
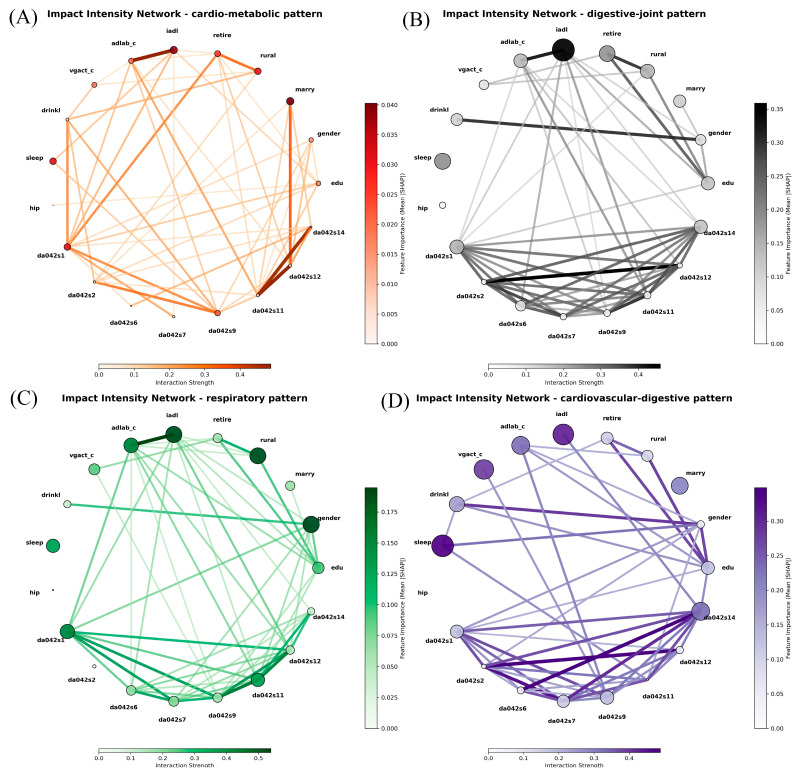
Impact-intensity network diagrams for the four multimorbidity patterns within the multimorbidity group. Pattern-specific synergistic interactions driving depressive symptoms. (**A**) Cardio-metabolic pattern; (**B**) digestive–joint pattern; (**C**) respiratory pattern; (**D**) cardiovascular–digestive pattern. Node size/color intensity = mean |SHAP| (feature importance). Edge thickness/color = interaction strength. These diagrams reveal clinically meaningful heterogeneity: cardio-metabolic patterns are more socially driven (e.g., marital status), digestive–joint patterns are pain-dominant, respiratory patterns show strong gender and multisite pain effects, and cardiovascular–digestive patterns are responsive to modifiable lifestyle factors (sleep and vigorous activity). See Methods 2.7 for detailed construction of the network diagrams.

To explore heterogeneity, we repeated the SHAP-based interpretability analysis on four literature-derived multimorbidity patterns within the multimorbidity group [6] (Figure 6). Distinct interaction networks emerged: (A) Cardio-metabolic pattern: The most important features (mean |SHAP value|) were marital status (0.0403), IADL disability (0.0385), headache (0.0319), sleep duration (0.0316), and rural residence (0.0310). (B) Digestive–joint pattern: IADL disability (0.3590), sleep duration (0.1909), retirement (0.1827), rural residence (0.1472), and headache (0.1462) were most influential, with strong IADL–ADL (interaction strength 0.4203) and shoulder pain-knee pain synergies (0.4586). (C) Respiratory pattern: gender (0.1947), IADL disability (0.1928), rural residence (0.1914), and ADL disability (0.1561) showed the highest impact and dense pain-related interactions. There are four pain-related patterns, with interaction strength from 0.3947 to 0.4685. (D) Cardiovascular–digestive pattern: sleep duration (0.3484), IADL disability (0.3153), vigorous activity (0.2824), and ADL disability (0.2351) were central, with prominent education–rural/gender/retirement (interaction strength 0.3850/0.3817/0.3783) and gender–drinking (0.3974) interactions.

These pattern-specific networks demonstrate that the drivers of depressive symptoms vary meaningfully across multimorbidity clusters, providing clinically actionable insights for targeted interventions.

## 4. Discussion

This cross-sectional analysis of the 2020 CHARLS wave revealed a significantly higher prevalence of depressive symptoms among middle-aged and older Chinese adults with multimorbidity (42.96%) compared to those with zero or one chronic condition (27.01%). Interpretable machine learning models demonstrated clinically acceptable discrimination (external AUROC of 0.7545 for the multimorbidity group and 0.7387 for the control group) and excellent calibration. The Gaussian process classifier (GPC) and generalized additive model (GAM) emerged as the best-performing models, respectively. SHAP-based interpretability consistently ranked functional disability (IADL and ADL), abnormal sleep duration, rural residence, and lower educational attainment as the primary contributors to depressive risk across the overall multimorbidity cohort, while vigorous physical activity and certain drinking patterns exhibited protective effects [23,24].

To further elucidate heterogeneity, we conducted pattern-specific SHAP analyses on four literature-derived multimorbidity clusters within the multimorbidity group. These analyses revealed distinct driver profiles and interaction networks (Figure 6), demonstrating that the pathways linking chronic disease clusters to depressive symptoms are not uniform but cluster-specific [17]. In the cardio-metabolic pattern, marital status emerged as the leading contributor (mean |SHAP| = 0.0403), followed by IADL disability, headache, sleep duration, and rural residence [25]. The strongest interactions involved IADL–ADL disability and shoulder pain–knee pain, suggesting that social isolation (unmarried/non-cohabiting status) synergistically amplifies functional decline and cephalic pain in patients with hypertension, diabetes, and dyslipidemia [26]. In contrast, the digestive–joint pattern was dominated by IADL disability (0.3590), sleep duration, retirement status, rural residence, and headache, with exceptionally strong synergies between shoulder–knee pain (0.4586) and IADL–ADL disability (0.4203). This pattern highlights a vicious cycle wherein joint pain and digestive complaints exacerbate mobility limitations, particularly among retired rural residents [27].

The respiratory pattern showed a distinct profile, with gender (0.1947), IADL disability, rural residence, and ADL disability ranking highest. Dense pain-related interactions (strength of 0.3947–0.4685) further underscored the role of multisite pain in lung disease patients. Finally, in the cardiovascular–digestive pattern, sleep duration (0.3484) and IADL disability (0.3153) were central, but vigorous physical activity exerted a significantly stronger protective effect (0.2824) [28,29]. This was accompanied by prominent interactions involving education with rural residence, gender, and retirement status, as well as gender with alcohol consumption (0.3850, 0.3817, and 0.3974). These findings indicate that modifiable lifestyle factors, such as physical activity and alcohol use, can mitigate risk more effectively in this cluster than in others [30]. Collectively, the pattern-specific networks reveal that while functional disability [31] and sleep disturbance [32] are universal drivers, their relative importance and synergistic partners vary meaningfully by multimorbidity phenotype: cardio-metabolic patterns are more socially driven; digestive–joint patterns are pain-dominant; respiratory patterns are influenced by gender and pain; and cardiovascular–digestive patterns are responsive to lifestyle factors.

Our findings extend previous CHARLS-based studies that reported associations between multimorbidity and depression using conventional regression approaches. By applying SHAP-based interpretability and pattern stratification, the present study moves beyond statistical associations to quantify feature importance, directional effects, and synergistic interactions. The observed pattern-specific heterogeneity helps explain why generic multimorbidity interventions often produce only modest mental health benefits, as one-size-fits-all strategies overlook cluster-specific mechanisms.

The superior performance of GPC and GAM, combined with decision-curve analysis demonstrating a net clinical benefit across a wide range of threshold probabilities, underscores the practical utility of these interpretable models [33]. Unlike black-box algorithms, the SHAP-driven, pattern-stratified approach allows clinicians to visualize how individual risk profiles are shaped by modifiable factors (sleep, activity, and pain management) versus non-modifiable ones (rural residence, education), enabling precision targeting within specific multimorbidity phenotypes [34]. This transparency is particularly valuable for shared decision-making and resource allocation in primary-care settings serving multimorbid older adults. Although the champion models achieved moderate discriminative performance (external AUROC 0.7545 in the multimorbidity group and 0.7387 in the control group), this level is comparable to or exceeds many recently published depression prediction models in middle-aged and older Chinese populations, which typically report AUROCs in the range of 0.70–0.82 [9,35,36]. Importantly, most prior studies focused on general older adult populations or single chronic conditions rather than multimorbidity patterns. Combined with excellent calibration (Brier scores ≈ 0.20) and positive net clinical benefit across wide threshold ranges in decision curve analysis, the models retain practical utility for risk stratification, triage, and prioritization of limited mental health resources in primary care—particularly in resource-constrained rural settings—rather than as standalone high-stakes diagnostic tools. We explicitly acknowledge that this performance may not suffice for autonomous clinical decision-making without clinician oversight or additional biomarkers or longitudinal data.

While visual inspection of SHAP summary plots and impact-intensity networks (Figure 5 and Figure 6) revealed clinically meaningful heterogeneity in feature importance and interactions across multimorbidity patterns, formal statistical testing of differences in SHAP value distributions (e.g., bootstrap CI overlap or permutation tests) was not performed due to computational demands and the primary exploratory aim. These distinct signatures (e.g., marital status prominence in cardio-metabolic vs. pain synergies in digestive–joint) provide strong face validity and warrant future dedicated comparative analyses.

Clinically, our results support integrating routine depressive-symptom screening (using CES-D-10) with brief functional (ADL/IADL) and sleep assessments into primary care and family doctor services, particularly for older adults with multimorbidity in rural China. Risk stratification by empirically derived multimorbidity patterns can help prioritize limited mental health resources: for example, unmarried individuals in cardio-metabolic clusters may benefit most from social support interventions, while those in digestive–joint clusters may gain from targeted pain management and mobility programs. Community-based exercise initiatives to improve IADL/ADL and promote vigorous activity appear especially promising for the cardiovascular–digestive cluster. From a policy perspective, the persistent rural–urban gradient and phenotype-specific drivers underscore the need for equity-focused resource allocation, including strengthened community mental health services and health insurance reforms that incentivize multimorbidity phenotyping and integrated care models. Targeted, phenotype-specific interventions—such as community-based exercise programs to improve IADL/ADL and vigorous activity in the cardiovascular–digestive cluster, pain-management protocols addressing shoulder–knee synergy in the digestive–joint cluster [37], or social-support enhancement for unmarried cardio-metabolic patients [38]—could more effectively interrupt the disability–depression cycle. From a policy perspective, the persistent rural–urban gradient and pattern-specific drivers call for strengthened mental health resources in underserved regions and equity-focused health insurance reforms that incorporate multimorbidity phenotyping [39].

Our research has the following strengths and weaknesses. Key strengths include: the use of a large national survey sample from which participants with missing data on depressive symptoms or key covariates were excluded, resulting in a final analytic sample with complete data for all modeled variables; rigorous feature selection (LASSO + Boruta + univariate screening with VIF < 5) and systematic comparison of 15 models using a composite AB Score; and the novel integration of pattern-specific SHAP, which advance the field toward precision public mental health [40]. Nevertheless, several limitations must be acknowledged. Although CHARLS employs a multi-stage stratified sampling design with available survey weights, the present analysis did not apply sampling weights. This choice was made to maintain consistency with prior CHARLS-based machine learning studies and to prioritize direct interpretability of associations in the analytic sample; therefore, while the sample is nationally representative in design, population-level inferences should be drawn with caution. The cross-sectional design precludes causal inference. Several key predictors (e.g., sleep disturbance, functional disability, and depressive symptoms) may have bidirectional relationships, and reverse causality cannot be excluded. Future longitudinal studies are needed to establish temporality. All chronic conditions and depressive symptoms were self-reported, introducing potential recall and social-desirability biases. Although CES-D-10 is well-validated in Chinese older adults, it is a screening tool rather than a clinical diagnosis [41]. Unmeasured confounders (e.g., cognitive function, social support quality, or inflammatory biomarkers) may have influenced results. Finally, although the 2020 wave reflects pre-COVID-19 conditions, subsequent pandemic-related disruptions may have altered the observed associations.

Moreover, the cross-sectional design precludes definitive causal inference. Key predictors such as abnormal sleep duration and functional disability (ADL/IADL) likely operate within bidirectional feedback loops: poor sleep and disability can precipitate or worsen depressive symptoms, while depressive states can further impair sleep architecture and functional capacity through motivational deficits and psychomotor retardation. Our models capture robust associations but cannot disentangle temporality; future longitudinal studies with repeated measures are essential to elucidate causal pathways and optimal intervention windows.

Future longitudinal studies employing causal machine learning frameworks (e.g., double/debiased ML) are warranted to establish temporality and quantify intervention effects within each multimorbidity pattern. Integration of these pattern-stratified, interpretable models into digital health platforms (e.g., mobile apps linked to electronic health records) could enable real-time risk stratification and personalized decision support. Ultimately, our study demonstrates that interpretable machine learning, when combined with multimorbidity-pattern decomposition, not only enhances predictive accuracy but also generates clinically meaningful, phenotype-tailored knowledge for reducing the mental health burden of multimorbidity in aging China.

## 5. Conclusions

This nationwide analysis of the 2020 CHARLS cohort demonstrates that middle-aged and older adults with multimorbidity are at substantially increased risk of depressive symptoms. Explainable machine learning identified functional disability, sleep duration, rural residence, and educational attainment as the principal determinants of depression, while also revealing clinically meaningful differences across multimorbidity patterns. These findings support the use of interpretable machine learning to facilitate personalized risk assessment and inform targeted prevention and intervention strategies aimed at improving mental health outcomes in aging populations.

## Figures and Tables

**Table 1:** Participant characteristics in the 2020 wave.

Characteristics, n (%) ^1^	Multimorbidity Group (n = 8265)		*p*-Value ^2^	Control Group (n = 6716)		*p*-Value ^2^
	Negative	Depression		Negative	Depression	
**Age** (mean ± SD, years)	63.37 ± 8.81	64.01 ± 8.88	**<0.001**	60.06 ± 9.09	61.13 ± 9.24	**<0.001**
**Gender**						
Female	2145 (25.95)	2219 (26.85)	**<0.001**	2202 (32.79)	1150 (17.12)	**<0.001**
Male	2569 (31.08)	1332 (16.12)		2700 (40.20)	664 (9.89)	
**Residence**						
Rural	2477 (29.97)	2431 (29.41)	**<0.001**	2790 (41.54)	1267 (18.87)	**<0.001**
Urban	2237 (27.07)	1120 (13.55)		2112 (31.45)	547 (8.14)	
**Education level**						
Below primary school	1533 (18.55)	1812 (21.92)	**<0.001**	1605 (23.90)	884 (13.16)	**<0.001**
Primary school	1105 (13.37)	810 (9.80)		1087 (16.19)	424 (6.31)	
Middle school	1267 (15.33)	638 (7.72)		1364 (20.31)	373 (5.55)	
High school and above	809 (9.79)	291 (3.52)		846 (12.60)	133 (1.98)	
**Marital status**						
Married/cohabitated	4156 (50.28)	2847 (34.45)	**<0.001**	4416 (65.75)	1548 (23.05)	**<0.001**
Other	558 (6.75)	704 (8.52)		486 (7.24)	266 (3.96)	
**Retirement**	1228 (14.86)	433 (5.24)	**<0.001**	848 (12.63)	144 (2.14)	**<0.001**
**ADLs**						
Normal	3967 (47.99)	2191 (26.51)	**<0.001**	4574 (68.11)	1465 (21.81)	**<0.001**
Disability	747 (9.04)	1360 (16.45)		328 (4.88)	349 (5.20)	
**IADLs**						
Normal	4101 (49.62)	2260 (27.34)	**<0.001**	4613 (68.69)	1476 (21.98)	**<0.001**
Disability	613 (7.42)	1291 (15.62)		289 (4.30)	338 (5.03)	
**Sleep duration (h)**						
(0, 6]	2733 (33.07)	2583 (31.25)	**<0.001**	2371 (35.30)	1187 (17.67)	**<0.001**
(6, 8]	1668 (20.18)	778 (9.41)		2187 (32.56)	507 (7.55)	
>8	313 (3.79)	190 (2.30)		344 (5.12)	120 (1.79)	
**Physical exercise**	4335 (52.45)	3177 (38.44)	**<0.001**	4497 (66.96)	1655 (24.64)	0.509
**Smoking**	1244 (15.05)	746 (9.03)	**<0.001**	1569 (23.36)	451 (5.72)	**<0.001**
**Drinking**	1897 (22.95)	1046 (12.66)	**<0.001**	2219 (33.04)	594 (8.84)	**<0.001**
**Hip fracture**	20 (0.24)	47 (0.57)	**<0.001**	11 (0.16)	16 (0.24)	**<0.001**
**Hypertension**	2741 (33.16)	1978 (23.93)	**0.026**	682 (10.15)	231 (3.44)	0.211
**Diabetes**	1111 (13.44)	831 (10.05)	0.860	110 (1.64)	32 (0.48)	0.225
**Dyslipidaemia**	2070 (25.05)	1424 (17.23)	**0.001**	243 (3.62)	84 (1.25)	0.581
**Lung diseases**	913 (11.05)	868 (10.50)	**<0.001**	119 (1.77)	43 (0.64)	0.892
**Heart disease**	1470 (17.79)	1246 (15.08)	**<0.001**	101 (1.50)	47 (0.70)	0.188
**Stroke**	358 (4.33)	377 (4.56)	**<0.001**	29 (0.43)	16 (0.24)	0.195
**Arthritis**	2316 (28.02)	2343 (28.35)	**<0.001**	578 (8.61)	345 (5.14)	**<0.001**
**Liver disease**	507 (6.13)	427 (5.17)	0.071	53 (0.79)	18 (0.27)	0.752
**Kidney disease**	692 (8.37)	665 (8.05)	**<0.001**	67 (0.99)	30 (0.45)	0.381
**Cancer**	161 (1.95)	126 (1.52)	0.744	29 (0.43)	16 (0.24)	0.195
**Stomach illness**	1991 (24.09)	1886 (22.82)	**<0.001**	487 (7.25)	208 (3.10)	0.067
**Asthma**	370 (4.48)	390 (4.72)	**<0.001**	10 (0.15)	5 (0.07)	0.581

^1^ Percentages shown for negative and depression subgroups are proportions of the total group size (n = 8265 for multimorbidity; n = 6716 for control). ^2^ *p*-values compare depression vs. non-depression within each group.

## Data Availability

Data are available in a publicly accessible repository. The data from the 2011, 2013, 2015, 2018, and 2020 China Health and Retirement Longitudinal Study (CHARLS) are publicly available at https://charls.charlsdata.com/pages/data/111/en.html, accessed on 16 November 2023. Further inquiries can be directed to the corresponding author.

## Supplementary Materials

The following supporting information can be downloaded at: https://www.mdpi.com/article/10.3390/healthcare14152341/s1; Supplementary Table S1. STROBE Statement—Checklist of items that should be included in reports of cross-sectional studies; Supplementary Table S2. Operational definitions and CHARLS questionnaire items for the 12 chronic conditions; Supplementary Table S3. Final selected predictors for multimorbidity group; Supplementary Table S4. Final selected predictors for control group; Supplementary Table S5. Complete performance metrics of all 15 machine learning models (multimorbidity group); Supplementary Table S6. Complete performance metrics of all 15 machine learning models (control group); Supplementary Table S7. Sensitivity analysis of machine learning models without SMOTE (multimorbidity group); Supplementary Table S8. Sensitivity analysis of machine learning models without SMOTE (control group); Supplementary Figure S1. Confusion matrix for the comorbidity group and control group.

## Abbreviations

The following abbreviations are used in this manuscript:

SHAP	Shapley Additive exPlanations
DALYs	Disability-adjusted life years
LASSO	Least Absolute Shrinkage and Selection Operator
VIF	Variance inflation factor
LR	Logistic regression
KNN	K-nearest neighbors
NB	Naïve Bayes
DT	Decision Tree
SVM	Support vector machine
MLP	Multilayer perceptron
GPC	Gaussian process classifier
RF	Random forest
GBDT	Gradient boosting decision tree
RotF	Rotation forest
GAM	Generalized additive model
