# Supplementary material for: Explainable Machine Learning Reveals Pattern-Specific Drivers of Depression in Middle-Aged and Older Adults with Multimorbidity: A Nationwide Cross-Sectional Study from CHARLS
Source: Healthcare (Basel). 2026 Aug 1;14(15):2341. doi: 10.3390/healthcare14152341 (PMC13466120; doi:10.3390/healthcare14152341)
Supplement: Supplementary file 1 [file healthcare-14-02341-s001.zip › healthcare-4409239-supplementary.pdf]

Supplementary Table S1. STROBE Statement—Checklist of items that should be included in reports of cross-sectional studies

	Item No	Recommendation	Page No
**Title and abstract**	1	(*a*) Indicate the study's design with a commonly used term in the title or the abstract	1-2
		(*b*) Provide in the abstract an informative and balanced summary of what was done and what was found	2
**Introduction**			
Background/rationale	2	Explain the scientific background and rationale for the investigation being reported	3
Objectives	3	State specific objectives, including any prespecified hypotheses	3
**Methods**			
Study design	4	Present key elements of study design early in the paper	4
Setting	5	Describe the setting, locations, and relevant dates, including periods of recruitment, exposure, follow-up, and data collection	4
Participants	6	(*a*) Give the eligibility criteria, and the sources and methods of selection of participants.	4
		(*b*) For matched studies, give matching criteria and number of exposed and unexposed	-
Variables	7	Clearly define all outcomes, exposures, predictors, potential confounders, and effect modifiers. Give diagnostic criteria, if applicable	5
Data sources/ measurement	8*	For each variable of interest, give sources of data and details of methods of assessment (measurement). Describe comparability of assessment methods if there is more than one group	4
Bias	9	Describe any efforts to address potential sources of bias	10
Study size	10	Explain how the study size was arrived at	4
Quantitative variables	11	Explain how quantitative variables were handled in the analyses. If applicable, describe which groupings were chosen and why	5
Statistical methods	12	(*a*) Describe all statistical methods, including those used to control for confounding	5-6
		(*b*) Describe any methods used to examine subgroups and interactions	5-6
		(*c*) Explain how missing data were addressed	4
		(*d*) If applicable, describe analytical methods taking account of sampling strategy	5
		(*e*) Describe any sensitivity analyses	-
**Results**			
Participants	13*	(a) Report numbers of individuals at each stage of study—eg numbers potentially eligible, examined for eligibility, confirmed eligible, included in the study, completing follow-up, and analysed	4
		(b) Give reasons for non-participation at each stage	-
		(c) Consider use of a flow diagram	Fig 1
Descriptive data	14*	(a) Give characteristics of study participants (eg demographic, clinical, social) and information on exposures and potential confounders	5
		(b) Indicate number of participants with missing data for each variable of interest	5
Outcome data	15*	Report numbers of outcome events or summary measures	7
Main results	16	(a) Give unadjusted estimates and, if applicable, confounder-adjusted estimates and their precision (eg, 95% confidence interval). Make clear which confounders were adjusted for and why they were included	7-8
		(b) Report category boundaries when continuous variables were categorized	5

		(c) If relevant, consider translating estimates of relative risk into absolute risk for a meaningful time period	5-6
Other analyses	17	Report other analyses done—eg analyses of subgroups and interactions, and sensitivity analyses	5-7
**Discussion**			
Key results	18	Summarise key results with reference to study objectives	7-9
Limitations	19	Discuss limitations of the study, taking into account sources of potential bias or imprecision. Discuss both direction and magnitude of any potential bias	10
Interpretation	20	Give a cautious overall interpretation of results considering objectives, limitations, multiplicity of analyses, results from similar studies, and other relevant evidence	8-9
Generalisability	21	Discuss the generalisability (external validity) of the study results	7-11
**Other information**			
Funding	22	Give the source of funding and the role of the funders for the present study and, if applicable, for the original study on which the present article is based	11-12

*Give information separately for exposed and unexposed groups.

Note: An Explanation and Elaboration article discusses each checklist item and gives methodological background and published examples of transparent reporting. The STROBE checklist is best used in conjunction with this article (freely available on the Web sites of PLoS Medicine at http://www.plosmedicine.org/, Annals of Internal Medicine at http://www.annals.org/, and Epidemiology at http://www.epidem.com/). Information on the STROBE Initiative is available at http://www.strobe-statement.org.

Supplementary Table S2. Operational definitions and CHARLS questionnaire items for the 12 chronic conditions

No.	Chronic Condition (English)	Exact CHARLS Questionnaire Item (DA007)	Notes
1	Hypertension	Have you been diagnosed with **Hypertension** by a doctor?	-
2	Dyslipidaemia	Have you been diagnosed with **Dyslipidemia** (elevation of low density lipoprotein, triglycerides, and total cholesterol, or a low high density lipoprotein level) by a doctor?	-
3	Diabetes	Have you been diagnosed with **Diabetes or high blood sugar** by a doctor?	Includes impaired glucose tolerance and elevated fasting blood glucose
4	Cancer	Have you been diagnosed with **Cancer or malignant tumor** (excluding minor skin cancers) by a doctor?	Excludes minor skin cancers
5	Lung diseases	Have you been diagnosed with **Chronic lung diseases**, such as chronic bronchitis or emphysema (excluding tumors or cancer) by a doctor?	-
6	Liver disease	Have you been diagnosed with **Liver disease** (except fatty liver, tumors, and cancer) by a doctor?	Excludes fatty liver, tumors, cancer
7	Heart disease	Have you been diagnosed with **Heart attack, coronary heart disease, angina, congestive heart failure, or other heart problems** by a doctor?	-
8	Stroke	Have you been diagnosed with **Stroke** by a doctor?	-
9	Kidney disease	Have you been diagnosed with **Kidney disease** (except for tumor or cancer) by a doctor?	Excludes tumor or cancer
10	Stomach / digestive disease	Have you been diagnosed with **Stomach or other digestive disease** (except for tumor or cancer) by a doctor?	Excludes tumor or cancer
11	Arthritis	Have you been diagnosed with **Arthritis or rheumatism** by a doctor?	-
12	Asthma	Have you been diagnosed with **Asthma** by a doctor?	-

Supplementary Table S3. Final selected predictors for multimorbidity group

Variable name	Variable Explanation	Univariate logistic	LASSO	Boruta	Selected
age	Age at the time of the interview				No
edu	Level of education				Yes
gender	Gender of respondents				Yes
marry	Marital status of respondents				Yes
rural	Respondents' usual place of residence (binary classification: rural or urban)				Yes
ins	Does the respondent have public health insurance (excluding private insurance)?				No
pension	Does the respondent have pension insurance (excluding commercial insurance)?				No
retire	Has the respondent already retired?				
family_size	Number of family members per respondent				No
hchild	Number of living children of the respondents				No
iadl	IADL (difficulty with 5 activities)				Yes
adlab_c	ADL (difficulty with 6 activities)				Yes
vgact_c	Do respondents engage in vigorous exercise each week?				Yes
mdact_c	Do respondents engage in moderate-intensity exercise each week?				No
ltact_c	Do respondents engage in light exercise each week?				No
exercise	Do respondents exercise every week?				No
drinkl	Does the respondent currently drink alcohol?				Yes
smokev	Did the respondent previously smoke?				No
smoken	Does the respondent currently smoke?				No
sleep	Average daily sleep duration of respondents				Yes
hip	Is there a hip fracture?				Yes
da042s1	Do you have a headache?				Yes
da042s2	Do you have shoulder pain?				Yes
da042s3	Do you have arm pain?				No
da042s4	Do you have wrist pain?				No
da042s5	Do your fingers hurt?				No
da042s6	Do you have chest pain?				Yes
da042s7	Do you have a stomachache?				Yes
da042s8	Do you have back pain?				No
da042s9	Do you have lower back pain?				Yes
da042s10	Do you have hip pain?				No
da042s11	Do you have leg pain?				Yes
da042s12	Do you have knee pain?				Yes
da042s13	Do you have ankle pain?				No
da042s14	Do your toes hurt?				Yes
da042s15	Do you have neck pain?				No
act_1	Social activities: visiting friends and socializing				No
act_2	Social activities: playing mahjong, playing chess, playing cards, visiting the community activity room				No
act_3	Social Activities: Providing assistance to relatives, friends, or neighbors who do not live with you				No
act_4	Social activities: Going to a park or other venues to				No

	dance, work out, practice qigong, etc.				
act_5	Social Activities: Participating in club activities				No
act_6	Social activities: Volunteer work or charity events				No
act_7	Social activities: Going to school or taking a training course				No
act_8	Social Activities: Other Social Activities				No

Green box: Selected; red box: Not selected; yellow box: Tentative

Supplementary Table S4. Final selected predictors for control group

Variable name	Variable Explanation	Univariate logistic	LASSO	Boruta	Selected
age	Age at the time of the interview				No
edu	Level of education				Yes
gender	Gender of respondents				Yes
marry	Marital status of respondents				Yes
rural	Respondents' usual place of residence (binary classification: rural or urban)				Yes
ins	Does the respondent have public health insurance (excluding private insurance)?				No
pension	Does the respondent have pension insurance (excluding commercial insurance)?				No
retire	Has the respondent already retired?				Yes
family_size	Number of family members per respondent				No
hchild	Number of living children of the respondents				No
iadl	IADL (difficulty with 5 activities)				Yes
adlab_c	ADL (difficulty with 6 activities)				Yes
vgact_c	Do respondents engage in vigorous exercise each week?				Yes
mdact_c	Do respondents engage in moderate-intensity exercise each week?				No
ltact_c	Do respondents engage in light exercise each week?				No
exercise	Do respondents exercise every week?				No
drinkl	Does the respondent currently drink alcohol?				Yes
smokev	Did the respondent previously smoke?				No
smoken	Does the respondent currently smoke?				No
sleep	Average daily sleep duration of respondents				Yes
hip	Is there a hip fracture?				No
da042s1	Do you have a headache?				Yes
da042s2	Do you have shoulder pain?				Yes
da042s3	Do you have arm pain?				No
da042s4	Do you have wrist pain?				No
da042s5	Do your fingers hurt?				Yes
da042s6	Do you have chest pain?				No
da042s7	Do you have a stomachache?				Yes
da042s8	Do you have back pain?				No
da042s9	Do you have lower back pain?				Yes
da042s10	Do you have hip pain?				No
da042s11	Do you have leg pain?				Yes
da042s12	Do you have knee pain?				Yes
da042s13	Do you have ankle pain?				No
da042s14	Do your toes hurt?				No
da042s15	Do you have neck pain?				No
act_1	Social activities: visiting friends and socializing				No
act_2	Social activities: playing mahjong, playing chess, playing cards, visiting the community activity room				No
act_3	Social Activities: Providing assistance to relatives, friends, or neighbors who do not live with you				No
act_4	Social activities: Going to a park or other venues to				

	dance, work out, practice qigong, etc.				
act_5	Social Activities: Participating in club activities				No
act_6	Social activities: Volunteer work or charity events				No
act_7	Social activities: Going to school or taking a training course				No
act_8	Social Activities: Other Social Activities				No

Green box: Selected; red box: Not selected; yellow box: Tentative

Supplementary Figure S1. Confusion matrix for the comorbidity group and control group

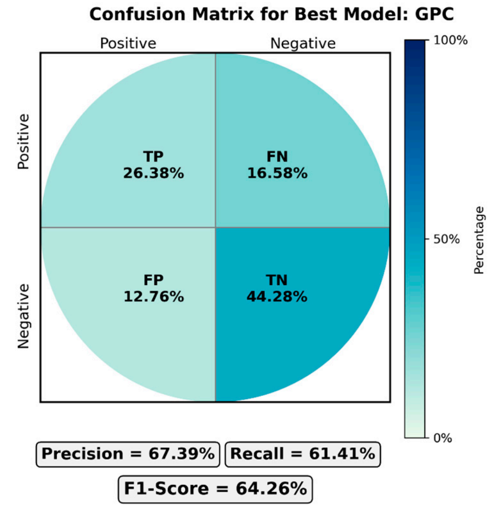

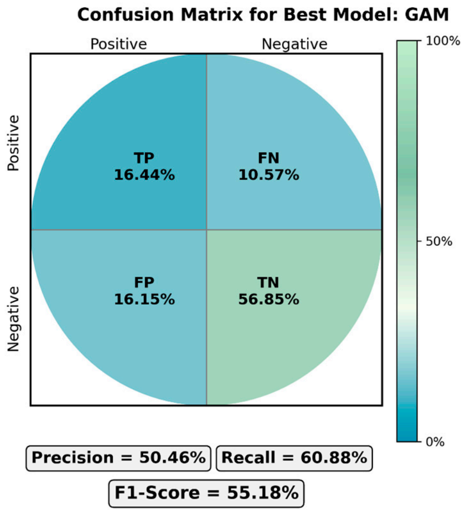

Left: Comorbidity group

Right: Control group

Supplementary Table S5. Complete performance metrics of all 15 machine learning models (multimorbidity group)

Model	AUC_Internal	Brier_Internal	AB_Score_Internal	Sensitivity_Internal	Specificity_Internal	F1_Internal	Accuracy_Internal	Youden_Internal
GPC	0.744188	0.200718	0.771735	0.594861	0.754972	0.619615	0.686177	0.349833
LightGBM	0.74396	0.200931	0.771515	0.739176	0.6131	0.65625	0.667272	0.352276
GBDT	0.743976	0.200973	0.771501	0.713481	0.646778	0.653871	0.675439	0.360259
GAM	0.743972	0.20081	0.771581	0.702218	0.653142	0.649414	0.674229	0.35536
ExtraTrees	0.737992	0.203402	0.767295	0.621964	0.724476	0.625819	0.68043	0.34644
RF	0.739806	0.202765	0.768521	0.646251	0.706179	0.634745	0.68043	0.35243
LR	0.741754	0.201416	0.770169	0.652235	0.703262	0.637537	0.681337	0.355497
XGBoost	0.73731	0.204074	0.766618	0.721577	0.626094	0.65069	0.66712	0.347671
AdaBoost	0.735528	0.210145	0.762692	0.727209	0.614161	0.649481	0.662734	0.341369
SVM	0.722505	0.20778	0.757363	0.712425	0.622381	0.643664	0.661071	0.334807
DT	0.696685	0.21608	0.740303	0.522703	0.758154	0.56701	0.656987	0.280858
RotF	0.701454	0.22129	0.740082	0.620204	0.689737	0.610428	0.659861	0.309942
NB	0.725585	0.283334	0.721125	0.671243	0.660037	0.632504	0.664852	0.33128
KNN	0.65673	0.260583	0.698074	0.502288	0.738531	0.543205	0.637024	0.240819
MLP	0.647819	0.278008	0.684905	0.575502	0.645452	0.562532	0.615396	0.220954
Model	AUC_External	Brier_External	AB_Score_External	Sensitivity_External	Specificity_External	F1_External	Accuracy_External	Youden_External
GPC	0.754549	0.198146	0.778202	0.614085	0.776246	0.642594	0.706594	0.390331
LightGBM	0.752711	0.199175	0.776768	0.728169	0.645811	0.662396	0.681186	0.37398
GBDT	0.752297	0.199082	0.776608	0.714085	0.662778	0.660586	0.684815	0.376863
GAM	0.751189	0.200112	0.775538	0.687324	0.680806	0.651101	0.683606	0.36813
ExtraTrees	0.754009	0.19895	0.777529	0.635211	0.741251	0.641993	0.695705	0.376463
RF	0.751517	0.199447	0.776035	0.66338	0.709438	0.647423	0.689655	0.372818
LR	0.748871	0.200193	0.774339	0.657746	0.718982	0.647712	0.69268	0.376728
XGBoost	0.750388	0.199722	0.775333	0.721127	0.648993	0.659369	0.679976	0.370119
AdaBoost	0.739694	0.209289	0.765202	0.721127	0.635207	0.653895	0.672111	0.356334
SVM	0.740065	0.204224	0.76792	0.711268	0.656416	0.65627	0.679976	0.367683
DT	0.716378	0.209919	0.75323	0.566197	0.763521	0.602247	0.678766	0.329718
RotF	0.702398	0.221801	0.740298	0.625352	0.682927	0.611149	0.658197	0.308279
NB	0.747107	0.274454	0.736326	0.690141	0.687169	0.655518	0.688445	0.377309
KNN	0.668388	0.256968	0.70571	0.532394	0.743372	0.568421	0.652753	0.275767
MLP	0.682048	0.24935	0.716349	0.646479	0.639449	0.60835	0.642468	0.285927

Rank	Model	AB_Score_Avg
1	GPC	0.774968
2	LightGBM	0.774141
3	GBDT	0.774054
4	GAM	0.77356
5	ExtraTrees	0.772412
6	RF	0.772278
7	LR	0.772254
8	XGBoost	0.770976
9	AdaBoost	0.763947
10	SVM	0.762642
11	DT	0.746766
12	RotF	0.74019
13	NB	0.728726
14	KNN	0.701892
15	MLP	0.700627

Supplementary Table S6. Complete performance metrics of all 15 machine learning models (control group)

Model	AUC_Internal	Brier_Internal	AB_Score_Internal	Sensitivity_Internal	Specificity_Internal	F1_Internal	Accuracy_Internal	Youden_Internal
GAM	0.720651	0.172437	0.774107	0.569263	0.740882	0.50167	0.694527	0.310145
GPC	0.719063	0.172444	0.773309	0.557547	0.737567	0.491943	0.688943	0.295113
LR	0.717293	0.172717	0.772288	0.532047	0.75695	0.486146	0.696203	0.288997
GBDT	0.710833	0.17521	0.767811	0.6306	0.674573	0.502471	0.662695	0.305172
LightGBM	0.711165	0.174675	0.768245	0.647829	0.656465	0.502943	0.654133	0.304294
XGBoost	0.702861	0.177172	0.762844	0.582357	0.702372	0.488016	0.669955	0.284729
RF	0.706326	0.175213	0.765556	0.603032	0.697526	0.498292	0.672003	0.300559
ExtraTrees	0.702429	0.176481	0.762974	0.618884	0.676868	0.496681	0.661206	0.295752
AdaBoost	0.711982	0.192024	0.759979	0.545141	0.748279	0.489935	0.69341	0.29342
DT	0.663726	0.185312	0.739207	0.51275	0.737567	0.461538	0.676843	0.250317
RotF	0.670693	0.191156	0.739768	0.474156	0.769192	0.452037	0.689501	0.243347
NB	0.705586	0.246644	0.729471	0.677464	0.621525	0.501787	0.636634	0.298989
SVM	0.648751	0.182528	0.733111	0.452102	0.818669	0.465578	0.719657	0.270771
KNN	0.626312	0.221893	0.702209	0.587181	0.62331	0.450794	0.613552	0.210492
MLP	0.608925	0.228395	0.690265	0.492764	0.691405	0.423578	0.637751	0.184169
Model	AUC_External	Brier_External	AB_Score_External	Sensitivity_External	Specificity_External	F1_External	Accuracy_External	Youden_External
GAM	0.73872	0.167609	0.785556	0.608815	0.778797	0.55181	0.732887	0.387613
GPC	0.730461	0.1693	0.780581	0.608815	0.763507	0.541667	0.721726	0.372322
LR	0.729445	0.170154	0.779645	0.581267	0.784913	0.53758	0.729911	0.366181
GBDT	0.729667	0.170171	0.779748	0.661157	0.701325	0.535714	0.690476	0.362482
LightGBM	0.728284	0.17052	0.778882	0.672176	0.69419	0.538037	0.688244	0.366366
XGBoost	0.727103	0.170431	0.778336	0.639118	0.730887	0.540163	0.706101	0.370005
RF	0.722556	0.172575	0.774991	0.628099	0.732926	0.534584	0.704613	0.361025
ExtraTrees	0.723348	0.17273	0.775309	0.650138	0.712538	0.535755	0.695685	0.362676
AdaBoost	0.734608	0.189604	0.772502	0.619835	0.771662	0.554187	0.730655	0.391496
DT	0.6886	0.179566	0.754517	0.504132	0.764526	0.471042	0.694196	0.268658
RotF	0.671318	0.188687	0.741315	0.484848	0.802243	0.480218	0.716518	0.287091
NB	0.712134	0.230898	0.740618	0.694215	0.636086	0.518519	0.651786	0.330301
SVM	0.649715	0.181118	0.734298	0.443526	0.830785	0.466667	0.72619	0.274311
KNN	0.6286	0.213647	0.707477	0.567493	0.662589	0.457778	0.636905	0.230082
MLP	0.628974	0.219062	0.704956	0.509642	0.723751	0.45177	0.665923	0.233393

Rank	Model	AB_Score_Avg
1	GAM	0.779831
2	GPC	0.776945
3	LR	0.775967
4	GBDT	0.77378
5	LightGBM	0.773563
6	XGBoost	0.77059
7	RF	0.770273
8	ExtraTrees	0.769142
9	AdaBoost	0.76624
10	DT	0.746862
11	RotF	0.740542
12	NB	0.735044
13	SVM	0.733705
14	KNN	0.704843
15	MLP	0.697611

Supplementary Table S7. Sensitivity analysis of machine learning models without SMOTE (multimorbidity group)

Model	AUC_Internal	Brier_Internal	AB_Score_Internal	Sensitivity_Internal	Specificity_Internal	F1_Internal	Accuracy_Internal	Youden_Internal
GPC	0.757381	0.20038	0.7785	0.702201	0.679926	0.694466	0.691063	0.382127
ExtraTrees	0.753809	0.20212	0.775845	0.68576	0.687881	0.686488	0.68682	0.373641
RF	0.748231	0.203368	0.772432	0.666667	0.698754	0.677537	0.68271	0.36542
LightGBM	0.74827	0.203367	0.772451	0.652082	0.713604	0.672777	0.682843	0.365685
GBDT	0.74776	0.203612	0.772074	0.646248	0.720764	0.671257	0.683506	0.367011
GAM	0.746529	0.204	0.771264	0.70777	0.646778	0.686824	0.677274	0.354548
LR	0.745226	0.204403	0.770412	0.629276	0.730841	0.662942	0.680058	0.360117
XGBoost	0.742847	0.206104	0.768371	0.72978	0.625298	0.693548	0.677539	0.355078
SVM	0.74156	0.205874	0.767843	0.712278	0.659242	0.693878	0.68576	0.371519
AdaBoost	0.74235	0.212294	0.765028	0.70061	0.64227	0.680752	0.67144	0.34288
DT	0.711358	0.216655	0.747352	0.495094	0.793689	0.581983	0.644391	0.288783
RotF	0.734445	0.212615	0.760915	0.773535	0.551047	0.696098	0.662291	0.324582
NB	0.723571	0.295266	0.714153	0.662159	0.658711	0.661019	0.660435	0.32087
KNN	0.708515	0.24252	0.732997	0.690533	0.617078	0.66607	0.653805	0.307611
MLP	0.690737	0.258833	0.715952	0.72315	0.560329	0.66871	0.64174	0.283479
Model	AUC_External	Brier_External	AB_Score_External	Sensitivity_External	Specificity_External	F1_External	Accuracy_External	Youden_External
GPC	0.746353	0.203113	0.77162	0.683099	0.681866	0.648829	0.682396	0.364965
ExtraTrees	0.74764	0.203117	0.772262	0.670423	0.688229	0.643243	0.680581	0.358652
RF	0.750881	0.201696	0.774593	0.666197	0.718982	0.653315	0.69631	0.385179
LightGBM	0.749037	0.203002	0.773018	0.643662	0.725345	0.640954	0.69026	0.369007
GBDT	0.748977	0.203001	0.772988	0.647887	0.727466	0.644709	0.693285	0.375353
GAM	0.747232	0.203313	0.77196	0.695775	0.675504	0.654305	0.684211	0.371278
LR	0.745766	0.203549	0.771108	0.625352	0.753977	0.640693	0.69873	0.379329
XGBoost	0.747265	0.203413	0.771926	0.722535	0.654295	0.662363	0.683606	0.37683
SVM	0.739852	0.206583	0.766634	0.7	0.669141	0.654378	0.682396	0.369141
AdaBoost	0.73925	0.211567	0.763841	0.687324	0.672322	0.647644	0.678766	0.359646
DT	0.720358	0.211429	0.754465	0.649296	0.69035	0.630212	0.672716	0.339646
RotF	0.697024	0.226243	0.73539	0.73662	0.563097	0.635866	0.637629	0.299716
NB	0.747377	0.264379	0.741499	0.687324	0.69035	0.655034	0.68905	0.377674
KNN	0.677888	0.255253	0.711318	0.643662	0.640509	0.606906	0.641863	0.284171
MLP	0.679156	0.254838	0.712159	0.709859	0.584305	0.627646	0.638234	0.294165

Rank	Model	AB_Score_Avg
1	GPC	0.77506
2	ExtraTrees	0.774053
3	RF	0.773512
4	LightGBM	0.772735
5	GBDT	0.772531
6	GAM	0.771612
7	LR	0.77076
8	XGBoost	0.770149
9	SVM	0.767239
10	AdaBoost	0.764435
11	DT	0.750908
12	RotF	0.748153
13	NB	0.727826
14	KNN	0.722158
15	MLP	0.714055

Supplementary Table S8. Sensitivity analysis of machine learning models without SMOTE (control group)

Model	AUC_Internal	Brier_Internal	AB_Score_Internal	Sensitivity_Internal	Specificity_Internal	F1_Internal	Accuracy_Internal	Youden_Internal
GAM	0.770405	0.195808	0.787298	0.806427	0.573833	0.722413	0.69013	0.38026
ExtraTrees	0.767311	0.197472	0.784919	0.816628	0.548585	0.720117	0.682606	0.365213
GBDT	0.741433	0.205756	0.767839	0.690385	0.664116	0.681435	0.677251	0.354501
LightGBM	0.74133	0.205841	0.767744	0.673043	0.680694	0.675627	0.676868	0.353736
RF	0.7332	0.208764	0.762218	0.730681	0.612089	0.68978	0.671385	0.34277
XGBoost	0.742295	0.205563	0.768366	0.617189	0.725325	0.652467	0.671257	0.342515
LR	0.731252	0.209302	0.760975	0.65519	0.681459	0.66391	0.668324	0.336649
AdaBoost	0.730056	0.216104	0.756976	0.609793	0.718184	0.644735	0.663989	0.327978
GPC	0.748819	0.2033	0.77276	0.767916	0.599847	0.708387	0.683882	0.367763
SVM	0.743003	0.206135	0.768434	0.79342	0.552155	0.708011	0.672788	0.345575
RotF	0.772807	0.194762	0.789022	0.82938	0.555981	0.729639	0.69268	0.385361
DT	0.716269	0.214406	0.750931	0.594491	0.721755	0.63489	0.658123	0.316246
KNN	0.714535	0.247169	0.733683	0.713083	0.577659	0.667861	0.645371	0.290742
NB	0.697021	0.314276	0.691372	0.568477	0.697526	0.607688	0.633002	0.266004
MLP	0.741875	0.219517	0.761179	0.670237	0.687325	0.676013	0.678781	0.357562
Model	AUC_External	Brier_External	AB_Score_External	Sensitivity_External	Specificity_External	F1_External	Accuracy_External	Youden_External
GAM	0.695901	0.208073	0.743914	0.727273	0.592253	0.514119	0.62872	0.319526
ExtraTrees	0.69218	0.209809	0.741186	0.713499	0.589195	0.505366	0.622768	0.302693
GBDT	0.710877	0.208841	0.751018	0.641873	0.697248	0.521837	0.682292	0.339121
LightGBM	0.709762	0.209959	0.749902	0.625344	0.708461	0.518265	0.686012	0.333805
RF	0.717704	0.208696	0.754504	0.699725	0.636086	0.521561	0.653274	0.33581
XGBoost	0.698302	0.211334	0.743484	0.570248	0.749235	0.507353	0.700893	0.319483
LR	0.711517	0.210993	0.750262	0.625344	0.710499	0.519451	0.6875	0.335844
AdaBoost	0.721871	0.21596	0.752956	0.608815	0.760449	0.539683	0.719494	0.369264
GPC	0.686294	0.212364	0.736965	0.658402	0.620795	0.49076	0.630952	0.279197
SVM	0.684166	0.21535	0.734408	0.724518	0.577982	0.505769	0.61756	0.3025
RotF	0.63836	0.228833	0.704764	0.644628	0.603466	0.474645	0.614583	0.248094
DT	0.696372	0.217117	0.739627	0.639118	0.667686	0.5038	0.65997	0.306804
KNN	0.641316	0.225738	0.707789	0.600551	0.643221	0.468314	0.631696	0.243772
NB	0.718559	0.223987	0.747286	0.630854	0.713558	0.524628	0.69122	0.344412
MLP	0.582232	0.270173	0.656029	0.504132	0.651376	0.412162	0.611607	0.155508

Rank	Model	AB_Score_Avg
1	GAM	0.765606
2	ExtraTrees	0.763052
3	GBDT	0.759428
4	LightGBM	0.758823
5	RF	0.758361
6	XGBoost	0.755925
7	LR	0.755619
8	AdaBoost	0.754966
9	GPC	0.754862
10	SVM	0.751421
11	RotF	0.746893
12	DT	0.745279
13	KNN	0.720736
14	NB	0.719329
15	MLP	0.708604
